# Diminished Returns of Educational Attainment on Body Mass Index Among Latino Populations: Insights from UAS Data

**DOI:** 10.31586/gjeid.2024.1096

**Published:** 2024-11-21

**Authors:** Shervin Assari, Hossein Zare

**Affiliations:** 1 Marginalized-Related Diminished Returns (MDRs) Research Center, Los Angeles, CA, USA; 2 Department of Family Medicine, Charles R. Drew University of Medicine and Science, Los Angeles, CA, USA; 3 Department of Urban Public Health, Charles R. Drew University of Medicine and Science, Los Angeles, CA, USA; 4 Department of Health Policy and Management, Johns Hopkins Bloomberg School of Public Health, Baltimore, MD, United States; 5 School of Business, University of Maryland Global Campus (UMGC), Adelphi, MD, United States

**Keywords:** Obesity, Body Mass Index, Educational Attainment, Minorities’ Diminished Returns, Latino, Health Disparities, Structural Inequality

## Abstract

**Background:**

Educational attainment is a well-established predictor of physical health outcomes, including body mass index (BMI). However, according to the theory of Minorities’ Diminished Returns (MDRs), the health benefits of education tend to be weaker for ethnic minorities compared to non-Latino Whites, due to structural inequalities and social disadvantages.

**Objective:**

This study examines whether the association between educational attainment and BMI is weaker among Latino individuals compared to non-Latino individuals, in line with the MDRs framework.

**Methods:**

Data were drawn from the 2014 wave of the Understanding America Study (UAS), a nationally representative internet-based panel. Body mass index (BMI) was the outcome of interest. Linear regression models were used to analyze the association between educational attainment and BMI, with an interaction term for ethnicity to explore differences in the relationship between Latino and non-Latino people. Models were adjusted for age, sex, marital status, and labor market participation and results were presented as beta coefficients, p-values, and 95% confidence intervals (CIs).

**Results:**

Higher educational attainment was associated with lower BMI for both Latino and non-Latino participants (p < 0.001). However, the interaction between educational attainment and ethnicity was significant (p < 0.05), indicating that Latino individuals experienced smaller reductions in BMI because of higher education compared to non-Latino people.

**Conclusion:**

This study provides evidence of diminished returns from educational attainment on BMI among Latino individuals. These findings support the MDRs framework, suggesting that structural barriers may limit the health benefits of education for Latino populations. While education is a key determinant of physical and mental health, its benefits are not equitably distributed across ethnic groups. Structural inequalities, chronic stress, poor neighborhood environments, and adverse educational and occupational conditions likely contribute to this disparity. Addressing these underlying factors through targeted policy interventions is necessary to promote health equity for Latino populations.

## 1. Background

Educational attainment is recognized as one of the most significant social determinants of health [1–5]. Individuals with higher levels of education and more years of schooling tend to exhibit better health outcomes, including lower body mass index (BMI) [6–17]. This can be attributed to several factors, such as higher health literacy, better nutrition, greater access to resources, lower stress levels, and higher levels of physical activity [18–22]. Both the quantity and quality of education contribute to the maintenance and development of overall health and fitness [20,23]. Conversely, any deterioration in the quality or quantity of education can diminish these health benefits [24–28].

Numerous studies have demonstrated that each additional year of education is associated with incremental reductions in BMI and a decreased prevalence of obesity and overweight [18,29,30]. However, some research has questioned the universality of this protective effect, suggesting that education does not always serve as a buffer against health issues [31]. This reinforces the idea that education’s impact on health outcomes, such as BMI, may vary across different social contexts [32–36].

While education is generally protective against a wide range of adverse outcomes, the extent of its benefits is not experienced equally across diverse ethnic and social groups [37]. The theory of Minorities’ Diminished Returns (MDRs) [38,39] posits that the positive effects of socioeconomic resources—particularly educational attainment—are less pronounced for ethnic minorities than for non-Latino White populations. Even when marginalized groups achieve similar levels of education, they often experience fewer health benefits (e.g., lower BMI) due to structural inequalities. These disparities arise from inequities in education quality, job opportunities, and access to resources, which reflect broader societal inequalities that cannot simply be explained by poverty or low education levels [37,40–46].

Structural barriers in systems such as education, the labor market, banking, and law enforcement disproportionately affect minority populations, limiting the full returns of education. These systems, rooted in a history of social marginalization and oppression, reduce the capacity of minority groups to convert educational attainment into optimal health outcomes [37]. As a result, MDRs manifest as reduced protective effects of education on health outcomes, including BMI, in minority populations [37,47–56].

Existing research has demonstrated MDRs across various behavioral, economic, and health domains. MDRs have been observed in health behaviors, mental health, and physical health outcomes [57–69]. For example, while higher educational attainment typically leads to increased income and wealth for both White and Black individuals, the financial returns are consistently lower for Black individuals [45,70,71]. This discrepancy can be attributed to factors such as labor market discrimination, segregation, and unequal access to high-quality education, which often result in highly educated Black individuals working in less favorable jobs and attending lower-quality schools compared to their White counterparts [72–74].

Similarly, the protective effects of educational attainment on health-related behaviors (e.g., substance use) and mental health outcomes (e.g., depression [63,75], anxiety [76], and suicide [63,77–79]) are weaker for Latino and Black populations than for their White counterparts. Some studies have also shown that the health benefits of education—such as increased physical activity [80], reduced obesity rates [32–36], improved diet [81,82], lower prevalence of heart disease [83], and reduced mortality [84]—are consistently weaker for Black individuals compared to White individuals.

While MDRs have been extensively studied across a range of outcomes, limited research has explored how MDRs specifically affect BMI, particularly in Latino populations. This is a significant gap in the literature, as Latino people represent a large and growing segment of the U.S. population, and the number of highly educated Latino people is increasing. Understanding how the health returns of education differ between Latino and non-Latino groups is crucial for addressing both educational and health disparities. Although some studies have investigated MDRs in relation to BMI correlates [32–36], most focus on Black-White differences, underscoring the need for further research on Latino populations.

## 2. Methods

### 2.1. Design and Setting

The Understanding America Study (UAS) [39,85–88] is a national internet-based panel survey managed by the University of Southern California (USC). Its goal is to offer insights into the social, economic, and health-related issues of the U.S. population. The UAS collects a wide variety of background data from all participants, including information on well-being, retirement planning, personality traits, and cognitive functioning. These core surveys are administered either annually or biennially, alongside repeated BMI measurement ensuring participants are accustomed to completing online surveys.

### 2.2. Participants and Sampling

Panel members are selected using probability-based sampling from post-office delivery sequence files. To ensure inclusivity, individuals who lack internet access are provided with internet-enabled tablets and service, allowing broad participation. The UAS panel consists of about 10,000 participants, including nearly 5,000 individuals aged 50 years or older.

### 2.3. Eligibility and Analytical Sample

For this study, data were drawn from the first wave (2014) of the Understanding America Study (UAS), focusing exclusively on Latino and non-Latino White adult participants. Participants were included in the analysis if they had complete data on BMI, educational attainment, and ethnicity.

### 2.4. Study Measures

BMI was calculated using self-reported height and weight measurements (r12bmi). The formula used was weight (kg) divided by height (m2). BMI was treated as a continuous variable, with higher scores representing greater fat content. Ethnicity was self-identified as non-Latino or Latino. Marital status (unmarried vs. married) and labor force participation (working in the last week) were self-reported and dichotomous variables. Education was self-reported and measured as years of schooling at baseline.

### 2.5. Data Analysis

All analyses were performed in Stata 18.0. Chi-square tests were used to evaluate differences in categorical variables between ethnic groups. Linear regression models were then employed to investigate the relationship between educational attainment and BMI. Two models were analyzed: **Model 1** served as the baseline model and included education as the primary predictor, with ethnicity, age, and sex included as covariates. **Model 2** extended the baseline model by incorporating an interaction term between ethnicity and educational attainment to assess whether the relationship between education and BMI varied by ethnicity. The results of the regression analyses were presented in terms of beta coefficients, p-values, and 95% confidence intervals (CIs). The significance of the interaction term indicated differences in the effect of educational attainment on BMI between ethnic groups. A significant interaction term was interpreted as evidence of Minorities’ Diminished Returns (MDRs), demonstrating that the benefits of educational attainment for BMI were weaker among Latino populations compared to non-Latino populations.

### 2.6. Ethics

All participants were previously enrolled in the UAS panel and had provided consent for participation in UAS studies. The UAS study protocol was approved by the University of Southern California (USC) Institutional Review Board (IRB). Data was collected and analyzed anonymously.

## 3. Results

### 3.1. Descriptive Data, Overall

Table 1 provides descriptive statistics for a sample of 5,722 individuals, including the mean and standard deviation for age, education, and BMI, as well as categorical distributions for ethnicity, gender, marital status, and labor market participation.

The average age of participants is 49 years, with a standard deviation of 16 years. Participants have an average of 11.17 years of education (approximately equivalent to a high school education), with a standard deviation of 2.22 years. The average BMI in the sample is 28.94, which falls within the overweight range, with a standard deviation of 6.97, indicating variability in participants’ body mass.

Non-Latino individuals make up the majority of the sample, with 89.7% (n=5,131). Latino individuals account for 10.3% (n=591) of the sample. Men represent 43.8% (n=2,508) of the sample. Women account for 56.2% (n=3,214) of the sample. Not married participants make up 40.3% (n=2,307) of the sample. Married participants represent 59.7% (n=3,415). Not in the labor market participants make up 41.7% (n=2,384). In the labor market participants account for 58.3% (n=3,338), indicating that the majority of participants are engaged in the workforce.

### 3.2. Descriptive Data, By Ethnicity

This table also presents a comparison of demographic and health characteristics between non-Latino (n=5,131) and Latino (n=591) groups.

Non-Latino participants report more years of education on average (11.25 years, SD = 2.22) compared to Latino participants (10.55 years, SD = 2.09). This reflects a statistically significant difference in educational attainment between the two groups, with Latino people having slightly less education on average (*p* < *0.05*). Non-Latino participants have a slightly lower average BMI (28.87, SD = 6.90) compared to Latino adults (29.53, SD = 7.53). While the difference in BMI is statistically significant (*p* < *0.05*), both groups fall within the overweight range on average. Non-Latino people have a significantly higher average age (50.08 years, SD = 15.55) compared to Latino adults (37.65 years, SD = 13.68). This suggests that the Latino group is younger on average than the non-Latino group (*p* < *0.05*).

Among non-Latino participants, 45.0% are men and 55.0% are women. In the Latino group, 33.8% are men and 66.2% are women. This indicates a higher proportion of women in the Latino group compared to the non-Latino group, with the difference being statistically significant (*p* < *0.05*). A greater percentage of non-Latino participants are married (61.2%) compared to Latino participants (46.7%). Conversely, a higher proportion of Latino participants are unmarried (53.3%) compared to non-Latino participants (38.8%), and this difference is statistically significant (*p* < *0.05*). Among non-Latino adults, 58.0% are in the labor market, compared to 61.4% of Latino participants. Although Latino participants have a slightly higher labor market participation rate, the difference between the two groups is statistically significant (*p* < *0.05*).

### 3.3. Regression Results without Interaction Term

Table 2 presents the results of a regression analysis examining the relationship between various predictors (age, gender, marital status, employment status, Hispanic ethnicity, and years of education) and Body Mass Index (BMI), without including any interaction terms. Key findings include:

Hispanic ethnicity is significantly associated with a higher BMI (B = .682, p = .029), indicating that, on average, Hispanic individuals have a higher BMI compared to non-Hispanic individuals. There is a significant negative association between years of education and BMI (B = −.420, p < .001). This suggests that higher educational attainment is associated with lower BMI, meaning that as individuals’ educational levels increase, their BMI tends to decrease. Age is significantly and positively associated with BMI (B = .019, p = .004), indicating that BMI increases slightly as individuals age.

Gender (being female) does not show a significant association with BMI (B = −.021, p = .910), suggesting that gender does not have a notable effect on BMI in this model. Employment status (currently working) does not have a significant effect on BMI (B = −.006, p = .977), indicating that employment status is not a major factor in predicting BMI in this model.

Without the interaction term, the model shows that higher educational attainment is significantly associated with lower BMI, while being Hispanic is linked to higher BMI. Age also positively impacts BMI, while gender, marital status, and employment status do not have a significant effect on BMI. This model reflects the overall relationship between education, ethnicity, and BMI without considering the potentially different impacts of education by ethnicity, which will be explored in models that include interaction terms.

### 3.4. Regression Results with Interaction Term

Table 3 presents the results of a regression analysis examining the relationship between various predictors (age, gender, marital status, employment status, Hispanic ethnicity, and years of education) and Body Mass Index (BMI), including an interaction term between years of education and Hispanic ethnicity. Key findings include:

There is a significant negative association between years of education and BMI (B = −.452, p < .001). This suggests that higher educational attainment is associated with lower BMI. The interaction term between years of education and Hispanic ethnicity is positive and significant (B = .351, p = .014). This indicates that the protective effect of education on reducing BMI is weaker for Hispanic individuals compared to non-Hispanic individuals. As years of education increase, Hispanic individuals experience a smaller reduction in BMI compared to non-Hispanic individuals, consistent with the theory of Minorities’ Diminished Returns (MDRs). Age is positively associated with BMI (B = .018, p = .005), indicating that as individuals get older, their BMI tends to increase slightly. Gender (being female) does not show a significant association with BMI (B = −.033, p = .862), suggesting that, in this model, gender does not significantly influence BMI. Marital status (being married) shows a positive but non-significant relationship with BMI (B = .244, p = .202), indicating that being married may slightly increase BMI, though this effect is not statistically significant. Employment status (currently working) also does not have a significant impact on BMI (B = −.028, p = .891).

## 4. Discussion

The aim of this study was to explore whether the relationship between educational attainment and BMI differs between Latino and non-Latino individuals, utilizing data from the Understanding America Study (UAS) [39,85–88]. Grounded in the theory of Minorities’ Diminished Returns (MDRs), we hypothesized that while higher levels of education would be associated with lower BMI for both groups, the strength of this association would be weaker among Latino participants. The MDRs framework suggests that the positive effects of socioeconomic resources, such as education, are less pronounced for marginalized populations compared to their non-minority peers.

The findings confirmed our hypothesis, indicating that although greater educational attainment was linked to lower BMI for both Latino and non-Latino participants, the health benefits of education—specifically the reduction in BMI—were less pronounced for Latino people. This aligns with past research on Black and other marginalized people [32–36]. Our finding is in line with the MDRs framework, implying that structural challenges may hinder Latino individuals from fully benefiting from educational attainment in terms of physical health [89]. A recent study explored how educational attainment influences marital status, employment, and food insecurity among Latino and non-Latino adults. Using data from the 2022 National Health Interview Survey (NHIS) with 27,648 participants, the research employed structural equation modeling to assess the mediating roles of marital status and employment in the relationship between education and food insecurity. Results showed that education’s protective effects against food insecurity were weaker for Latino individuals due to higher unemployment rates and lower marriage rates. These findings highlight structural barriers that prevent Latino people from fully benefiting from educational attainment to improve their marital and employment outcomes, which in turn exacerbates food insecurity. The study calls for targeted interventions to address these disparities, emphasizing the need for a comprehensive approach beyond improving educational access to reduce food insecurity among Latino communities [89].

A robust body of research consistently shows that education positively influences health outcomes, including BMI [18,29,30]. Higher educational attainment is linked to better health literacy, improved nutrition, and greater access to resources that support healthy living, which collectively contribute to lower BMI [18,29,30]. Education also reduces stress and fosters problem-solving skills, providing individuals with the tools to navigate health challenges more effectively. Many studies have found that each additional year of schooling is associated with lower BMI and improved health outcomes [18,29,30], emphasizing the critical role that education plays in maintaining physical health throughout adulthood.

Extensive research on MDRs highlights that the protective effects of education and other socioeconomic resources tend to be weaker for ethnic minorities, particularly Black individuals [37,41,44,47,51,56,90–95]. Studies have demonstrated that while higher educational attainment generally leads to better outcomes such as higher income, better mental health, and improved physical health, these benefits are often smaller for Black individuals compared to Whites [45,72,96,97]. For instance, educated Black individuals frequently earn less and work in lower-quality jobs than their White peers, which diminishes the positive health effects of education [73,98,99]. Though much of the existing MDRs research has focused on Black populations, relatively little attention has been given to Latino individuals, especially in terms of how education affects physical health outcomes such as BMI.

To date, research exploring the diminished returns of education in Latino populations has been limited, and few studies have examined how educational attainment impacts health outcomes, such as BMI, within this group. While previous studies have acknowledged that Latino people face similar structural barriers to other marginalized populations—such as discrimination, limited economic opportunities, and unequal access to quality education—the direct impact of these factors on the health returns of education remains underexplored [100–103]. Our study contributes to this growing area of research, providing evidence that the health benefits of education, specifically with respect to BMI, are reduced for Latino individuals. This highlights the importance of further research on the MDRs affecting Latino populations, particularly in the realm of physical health.

Several factors may explain why the health benefits of education are diminished for Latino people. Structural inequality is likely a primary contributor, as systemic barriers limit access to high-quality education, healthcare, and economic opportunities, even for those with higher levels of education [104–106]. Latino individuals may also experience heightened chronic stress due to social marginalization, economic instability, and discrimination, all of which can adversely impact physical health over time [107–109]. Additionally, labor market discrimination may prevent Latino individuals from securing jobs that support a healthy lifestyle, reducing their ability to translate educational gains into better health [110–112]. Poor nutrition, higher food insecurity, and lower access to health-promoting resources further exacerbate these challenges, creating an environment in which the protective effects of education on BMI are diminished for Latino people [113,114].

### 4.1. Implications

The findings from this study have implications for public health and educational policy. Efforts to improve physical health among Latino populations should go beyond simply increasing access to education. Policymakers must address the structural barriers that prevent education from fully benefiting Latino individuals, such as labor market discrimination and limited access to high-quality jobs. Additionally, public health strategies that focus on improving nutrition, healthcare access, and social support systems could help mitigate the negative effects of chronic stress and economic insecurity. Tailored interventions that address these structural and social determinants of health are essential for improving health outcomes such as BMI in Latino populations.

### 4.2. Limitations

Several limitations should be considered when interpreting these findings. First, the study collected data on Latino participants, but we did not have information on country of origin or specific Latino subgroups [115]. Latinos are highly heterogeneous in terms of dietary patterns, socioeconomic status, cultural practices, and other factors that could influence both education and BMI. Additionally, the study sample was not nationally representative, which may limit the generalizability of the findings. While we controlled for demographic factors such as age and sex, other potential confounders—such as childhood socioeconomic conditions and early educational opportunities—were not accounted for in this analysis. Environmental factors, including neighborhood-level access to food resources, were also not measured. Furthermore, our analysis was restricted to Latino and non-Latino White individuals, excluding other marginalized groups and unexamined dimensions of marginalization, such as race, religion, and geographic location. BMI was self-reported, introducing potential measurement bias. Finally, the cross-sectional nature of the study limits our ability to infer causation in the relationship between education and BMI. Despite these limitations, the findings contribute valuable insights into ethnic variation in the returns of education on BMI. Future longitudinal studies with repeated measures of both education and BMI are needed to examine how the relationship between education and BMI may change over time and differ across ethnic groups.

## 5. Conclusion

This study adds further evidence to the theory of Minorities’ Diminished Returns (MDRs), demonstrating that the health benefits of education—specifically in relation to BMI—are less substantial for Latino individuals compared to their non-Latino counterparts. While education is a key determinant of health, its positive impact is not equally experienced across ethnic groups. Structural inequalities, chronic stress, labor market discrimination, residential segregation, and unequal access to nutritious food options (such as food deserts) likely contribute to the diminished health returns of education observed among Latino populations. Addressing these disparities calls for multi-level interventions that go beyond improving individual access to education and resources; it requires tackling systemic barriers that disproportionately affect minoritized and racialized groups. Only through such a comprehensive approach can we make strides toward health equity for marginalized communities.

## Figures and Tables

**Table 1.** Descriptive tables, overall and by ethnicity

	All (n = 5,722)		Non-Latino (n = 5,131)		Latino (n = 591)	
	Mean	Std. Deviation	Mean	Std. Deviation	Mean	Std. Deviation
Age (Yrs) *	49	16	50	15	38	14
Education Years*	11.17	2.216	11.25	2.218	10.55	2.093
BMI (Yr 12)*	28.93	6.97	28.87	6.90	29.53	7.53
	n	%	n	%	n	%
Ethnicity						
Non-Latino	5,131	89.7	5,131	100	-	-
Latino	591	10.3	-	-	591	100
Gender*						
Men	2,508	43.8	2,308	45.0	200	33.8
Women	3,214	56.2	2,823	55.0	391	66.2
Married*						
No	2,307	40.3	1,992	38.8	315	53.3
Yes	3,415	59.7	3,139	61.2	276	46.7
In Labor market*						
No	2,384	41.7	2,156	42.0	228	38.6
Yes	3,338	58.3	2,975	58.0	363	61.4

* p < 0.05 for comparison of Latino and non-Latino

**Table 2.** Summary of regression without the interaction

Model	Unstandardized Coefficients		Standardized Coefficients	Sig.	95.0% Confidence Interval for B	
	B	Std. Error	Beta		Lower Bound	Upper Bound
Age	.019	.007	.042	.004	.006	.032
Female	−.021	.188	−.002	.910	−.390	.348
Married	.248	.191	.017	.194	−.126	.623
Working	−.006	.205	.000	.977	−.408	.397
Hispanic	.682	.312	.030	.029	.070	1.294
Education (Years)	−.420	.042	−.134	.000	−.503	−.337

Dependent Variable: Body Mass Index (BMI)

**Table 3.** Summary of regression with the interaction

	Unstandardized Coefficients		Standardized Coefficients	Sig.	95.0% Confidence Interval for B	
	B	Std. Error	Beta		Lower Bound	Upper Bound
						
Age	.018	.007	.041	.005	.005	.031
Female	−.033	.188	−.002	.862	−.401	.336
Married	.244	.191	.017	.202	−.130	.618
Working	−.028	.205	−.002	.891	−.431	.375
Hispanic	−3.042	1.551	−.132	.050	−6.082	−.003
Education (Years)	−.452	.044	−.144	.000	−.539	−.365
Education (Years) x Hispanic	.351	.143	.165	.014	.070	.631
